# Incidental papillary fibroelastoma of the tricuspid valve

**DOI:** 10.1186/1749-8090-9-123

**Published:** 2014-07-10

**Authors:** Thomas Strecker, Sabine Scheuermann, Ehab Nooh, Michael Weyand, Abbas Agaimy

**Affiliations:** 1 Center of Cardiac Surgery, Friedrich-Alexander-University Erlangen-Nuremberg, Krankenhausstraße 12, 91054 Erlangen, Germany; 2 Department of Cardiology, Friedrich-Alexander-University Erlangen-Nuremberg, Krankenhausstraße 12, 91054 Erlangen, Germany; 3 Institute of Pathology, Friedrich-Alexander-University Erlangen-Nuremberg, Krankenhausstraße 12, 91054 Erlangen, Germany

**Keywords:** Cardiac tumors, Papillary fibroelastoma, Echocardiography, Cardiac surgery, Pathology

## Abstract

Primary cardiac tumors are very rare, papillary fibroelastoma (PFE) being the second most common benign tumor of the heart in previous series. However, as a consequence of increased imaging examinations, incidental PFE may represent the most common cardiac tumor. Their clinical presentation varies from incidental asymptomatic masses to severe life-threatening cardiovascular complications necessitating emergency surgery. Here we report the diagnostic evaluation and successful surgical resection of such a cardiac tumor in a 67-year-old woman. Histology confirmed diagnosis of a papillary fibroelastoma. This report demonstrates it’s necessary to include cardiac tumors in the differential diagnosis of subtle and non-specific cardiothoracic symptoms.

## Background

Primary cardiac tumors are very rare entities among heart disease. Their frequency in previous autopsy studies ranged from 0.001 to 0.03% [1,2]. About 75% of cardiac tumors are benign, atrial myxomas being the most common type [1,2]. Papillary fibroelastoma (PFE) is a cardiac tumor that predominantly originates from the heart valves, oftentimes from the aortic [3] or mitral valve [4]. This usually small lesion shows a characteristic echocardiographic, gross, and histological appearance [5].

Here we present the diagnostic evaluation and successful surgical resection of such a cardiac tumor which was found on a medical check-up in a 62-year-old patient with progressive chest pain. Histology confirmed the definite diagnosis of a papillary fibroelastoma.

## Case presentation

**Figure 1:**
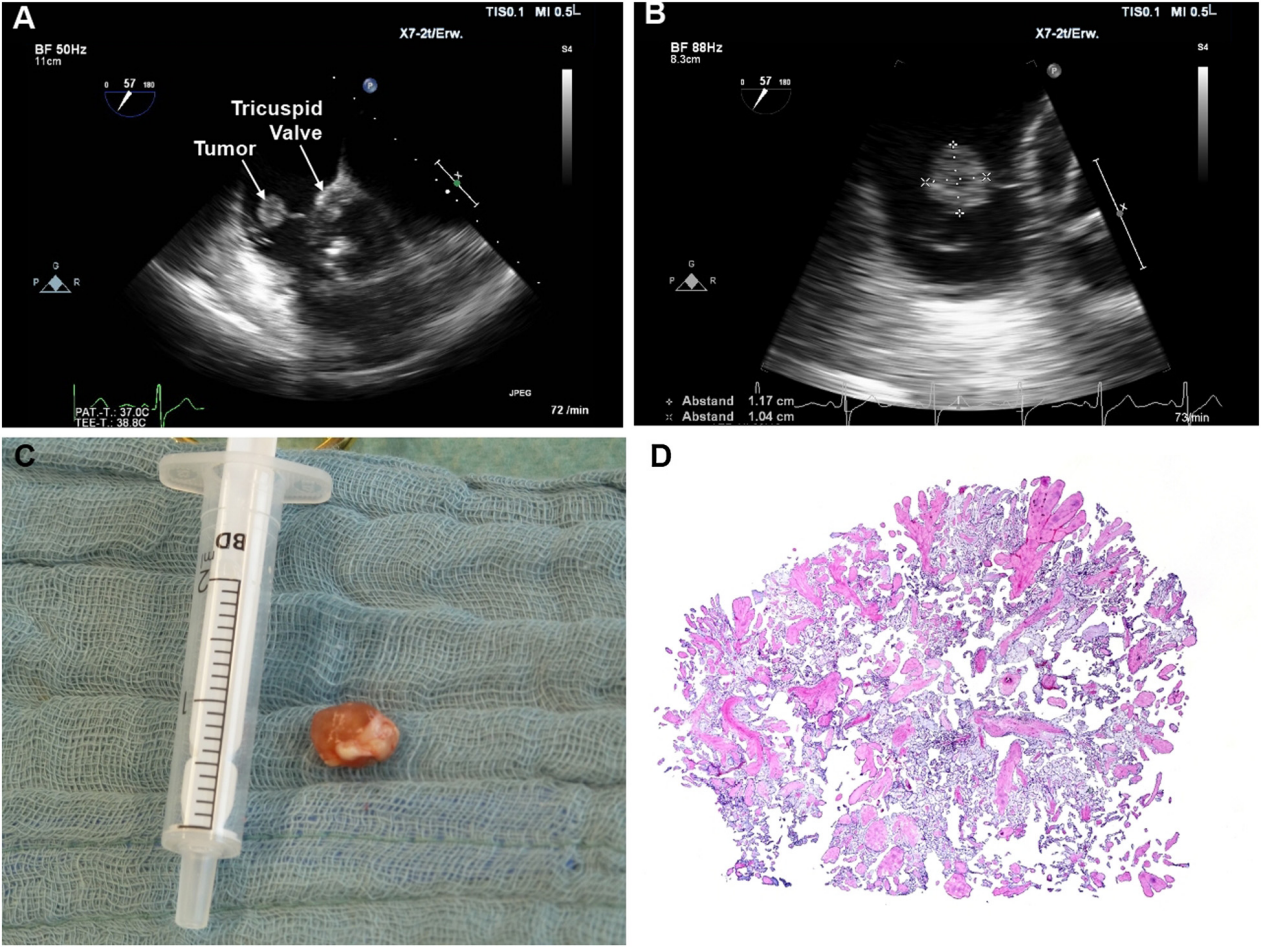
**Papillary fibroelasoma of the tricuspid valve. A**: Transesophageal echocardiography (TEE) depicted a nodular mobile structure at the anterior leaflet of the tricuspid valve. **B**: At higher magnification, TEE demonstrated a slight insufficient tricuspid valve and a tumor diameter of 10 × 11 mm. **C**: Intraoperative gross photograph of the tumor. **D**: Histological examination showed complex branching papillary fronds characteristic of papillary fibroelastoma.

A 67-year-old Caucasian woman with typical symptoms of angina pectoris was referred to the cardiological department of our hospital for further diagnostic evaluation. She noted progressive episodes of gnawing chest pain during the last weeks.At admission, the electrocardiogram (ECG) showed sinus rhythm with ST-decreases in I and AVF. Laboratory examination revealed a troponin I level of 2.3 ng/ml (normal range <0.5 ng/ml). A chest radiograph showed a normal heart configuration without pulmonary congestion, infiltration, effusion or pneumothorax. Transthoracic (TTE) and transesophageal echocardiography (TEE) demonstrated a normal left ventricular ejection fraction and no pericardial effusions. The heart valves appeared unremarkable. However, a slight tricuspid valve regurgitation with a small mobile nodular tumor mass at the anterior leaflet was seen (Figure 1, Panels A and B). Subsequent invasive coronary angiography revealed significant stenosis of all three main coronary arteries (left main, left circumflex and right coronary artery).The patient was taken to the operating theatre, where a median sternotomy was performed and cardiopulmonary bypass was installed via aorto-bicaval cannulation with normothermia. After opening of the right atrium, a soft friable gelatinous tumor was successfully excised (Figure 1, Panel C). Afterwards, the left internal mammary artery was anastomosed to the left anterior descending (LAD), and two single saphenous vein graphs were anastomosed to the ramus circumflex (RCX) and the right coronary artery (RCA). Patient’s recovery was uneventful. Histological examination of the resected tissue revealed a papillary fibroelastoma with complex branching papillary fronds and characteristic three-layered pattern of papillary fibroelastoma (Figure 1, Panel D).

## Conclusions

PFE represents the most common tumorous lesion of cardiac valves [5]. Few cases may arise from the atrial endocardium away from the valves and the valves leaflets [6]. Although most PFEs are less than 10 mm in diameter, rare giant examples have been reported [7]. The clinical symptoms of these tumors are very often non-specific, and they usually present themselves insidiously that their diagnostic and surgical management is often delayed [2]. Reported symptoms include arrhythmias, chest pain, dyspnea, syncope and pericardial effusions with tamponade as well as intracardiac blood flow obstruction, cerebrovascular and peripheral embolization or sudden death [6-8]. The observation that most PFEs arise in diseased cardiac valves or after a history of instrumentation or previous irradiation make it likely that an initial minute lesion (nidus) is necessary for further growth of the lesion [5]. Grossly, PFE strikingly resemble the appearance of a sea anemone upon immersion in water [3].

The radiological evaluation of these cardiac neoplasms has been greatly facilitated by the development of noninvasive cardiac imaging [9]. Although TTE is really useful in the initial evaluation of suspected cardiac masses, TEE is commonly required for a more comprehensive and accurate assessment [10].

In the majority of cases, these tumors require operative excision to prevent potentially life-threatening complications; only a few cases may be unresectable because of their large size, and only tumor debulking may be possible in such cases [2]. Although the long-term prognosis of asymptomatic tumors is often good, fatal histories have been reported for untreated symptomatic tumors. Therefore, if a cardiac tumor is found to cause symptoms, indication for operation should be liberal [2].

In summary, we described an unusual case of a PFE on the tricuspid valve.

## Consent

Written informed consent was obtained from the patient for publication of this Case report and any accompanying images. A copy of the written consent is available for review by the Editor-in-Chief of this journal.

## Competing interests

The authors declare that they have no competing interests.

## Authors’ contributions

All authors participated in the design of the case report and coordination and helped to draft the manuscript. All authors read and approved the final manuscript.
